# Zinner syndrome: report of a case and whole exome sequencing

**DOI:** 10.1186/s12610-025-00256-3

**Published:** 2025-03-11

**Authors:** Jiatai He, Chengcheng Wei, Yu Huang, Feixiang Xu, Miao Wang, Zhaohui Chen

**Affiliations:** 1 Department of Urology, Union Hospital, Tongji Medical College, Huazhong University of Science and Technology, Wuhan, Hubei 430074 China; 2 Department of Urology, The First Affiliated Hospital of Chongqing Medical University, Chongqing, 400016 China

**Keywords:** Zinner syndrome, Whole exome sequencing, Genitourinary malformation, Laparoscopic surgery, Syndrome de Zinner, Malformation génito-urinaire, Chirurgie laparoscopique

## Abstract

**Background:**

Zinner syndrome is a rare congenital malformation of the male genitourinary system, characterized by a triad: seminal vesicle cyst, unilateral renal agenesis, and ipsilateral ejaculatory duct obstruction. The etiology of this uncommon disease remains largely elusive; however, genetic mutations may contribute to its development. In this report, we present a case of symptomatic Zinner syndrome that was surgically treated, alongside an investigation into the potential genetic basis of the syndrome via whole exome sequencing.

**Case presentation:**

We report the case of an 18-year-old male presenting with urinary pain and was diagnosed with right renal agenesis and a left seminal vesicle cyst following comprehensive imaging. The patient also experienced perineal pain and urgency, without symptoms of frequent urination, dysuria, or hematuria, and no familial history of genitourinary anomalies was documented. He successfully underwent laparoscopic resection of a pelvic mass, with pathological examination confirming a seminal vesicle cyst. Postoperative recovery was uneventful. Whole exome sequencing of blood and tissue samples highlighted myeloma overexpressed gene (*MYEOV*), B melanoma antigen family member (*BAGE*), and N-acetylated-alpha-linked acidic dipeptidase 2 (*NAALAD2*) as potential mutated genes related to Zinner syndrome. Additionally, two predisposing genetic variants were identified.

**Conclusions:**

Zinner syndrome is a rare condition commonly diagnosed via various imaging modalities. Surgical resection remains the most effective treatment for symptomatic cases. Gene sequencing provides valuable insights into the genetic etiology of Zinner syndrome, enhancing our understanding and potentially guiding future diagnostic approaches.

**Supplementary Information:**

The online version contains supplementary material available at 10.1186/s12610-025-00256-3.

## Résumé

**Contexte.:**

Le syndrome de Zinner est une malformation congénitale rare de l’appareil génito-urinaire masculin, caractérisée par une triade : kyste d’une vésicule séminal, agénésie rénale unilatérale et obstruction du canal éjaculateur ipsilatéral. L’étiologie de cette pathologie peu fréquente reste largement insaisissable ; cependant, des mutations génétiques peuvent contribuer à son développement. Dans ce rapport, nous présentons un cas de syndrome de Zinner symptomatique qui a été traité chirurgicalement, ainsi qu’une enquête sur une potentielle base génétique de ce syndrome via le séquençage de l’exome entier.

**Présentation du cas.:**

Il s’agit d’un homme de 18 ans présentant une douleur urinaire et ayant reçu un diagnostic d’agénésie rénale droite et de kyste de la vésicule séminale gauche à la suite d’une imagerie complète. Le patient a également ressenti une douleur et une urgence périnéales, sans symptômes de mictions fréquentes, de dysurie ou d’hématurie ; aucun antécédent familial d’anomalies génito-urinaires n’a été documenté. Il a subi avec succès une résection laparoscopique d’une masse pelvienne, avec un examen pathologique confirmant un kyste vésiculaire séminal. La récupération postopératoire s’est déroulée sans incident. Le séquençage de l’exome entier d’échantillons de sang et de tissus a mis en évidence le gène surexprimé du myélome (MYEOV), le membre de la famille de l’antigène du mélanome B (BAGE) et la dipeptidase acide 2 (NAALAD2) liée à la N-acétylée alpha comme gènes potentiels liés au syndrome de Zinner. De plus, deux variants génétiques prédisposants ont été identifiées.

**Conclusions.:**

Le syndrome de Zinner est une maladie rare habituellement diagnostiquée via diverses modalités d’imagerie. La résection chirurgicale reste le traitement le plus efficace pour les cas symptomatiques. Le séquençage des gènes fournit des informations précieuses sur l’étiologie génétique du syndrome de Zinner, améliorant ainsi notre compréhension et guidant potentiellement les futures approches diagnostiques.

## Introduction

Zinner syndrome is characterized by a triad of mesonephric (Wolffian) duct malformations, including unilateral renal agenesis, ipsilateral seminal vesicle cyst, and ejaculatory duct obstruction. It is often regarded as the male counterpart to Mayer-Rokitansky-Küster-Hauser (MRKH) syndrome, which involves congenital agenesis of the vagina and uterus in females [1]. The etiology of Zinner syndrome lies in the abnormal development of the mesonephric duct and the absence of ureteral buds during embryogenesis [2]. Individuals with Zinner syndrome typically remain asymptomatic until engaging in sexual activity. However, they may experience a range of symptoms before the age of 40, including hematuria, urinary difficulties, urgency, and pain during ejaculation [3, 4].

Embryogenesis can be influenced by various factors leading to structural anomalies. Notably, ionizing radiation and certain medications have teratogenic effects [5]. The advent of large-scale parallel sequencing technologies, such as whole exome sequencing (WES), has expanded the landscape for discovering genetic causes of rare diseases [6]. WES is adept at identifying variations within protein-coding regions across genes and is an effective method for detecting copy number variations (CNVs) in potential candidate genes, given that most disease-causing mutations occur in exons [7]. In this study, we performed WES on blood and tissue samples from the patient to investigate the potential link between genetic variations and Zinner syndrome.

## Case presentation

An 18-year-old male presented to our hospital with a two-week history of perineal pain, accompanied by urinary urgency and pain, but without ejaculatory pain, dysuria, or hematospermia. Routine laboratory investigations were unremarkable. Physical examination revealed a generally healthy status, with a soft abdomen devoid of palpable masses. The patient's family history was negative for Zinner syndrome, and his parents were asymptomatic and had not undergone any related medical evaluations. Furthermore, the union between his parents is devoid of consanguineous relationships.

**Fig. 1:**
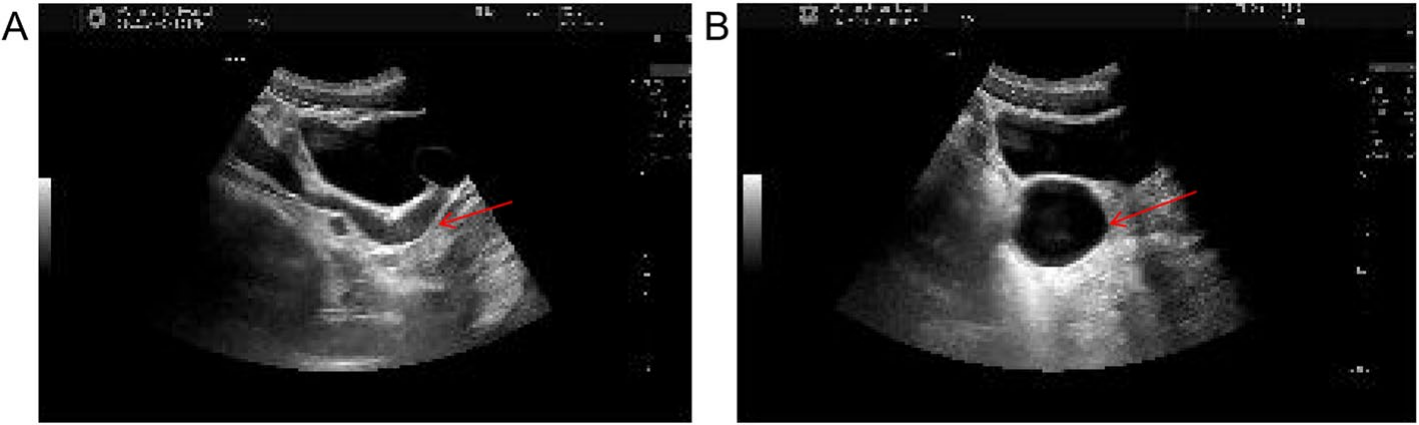
Ultrasonographic findings in the patient. **A** An anechoic region indicative of an abnormality was shown along the mid-lower ureteral pathway, as highlighted by the red arrow. **B** A cystic anechoic area measuring 57.5 × 44.5 mm was presented near the left seminal vesicle gland, also marked by a red arrow

**Fig. 2:**
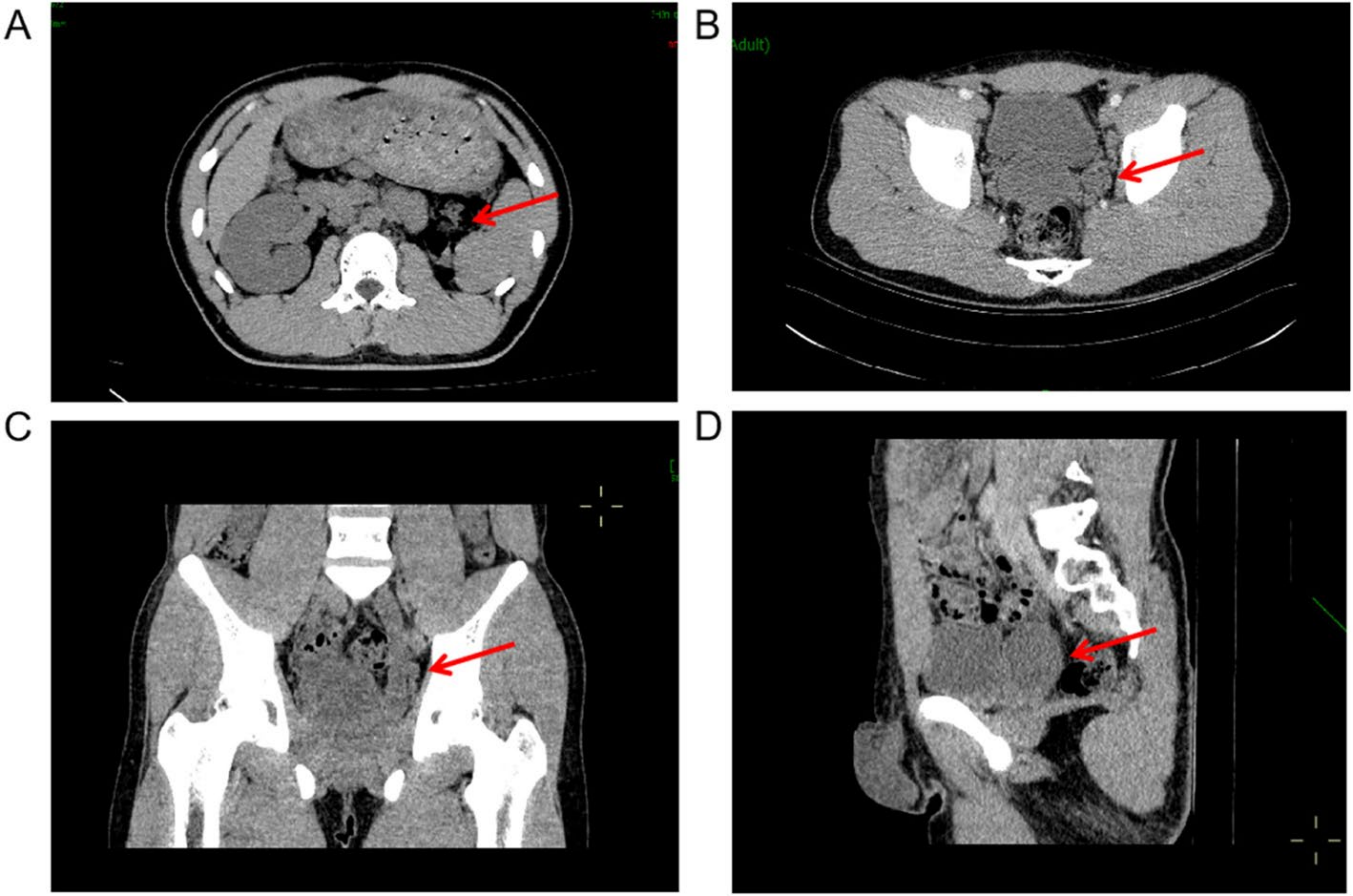
Computed tomography findings in the patient. **A** An axial abdominal CT plain scan highlighted the absence of the left kidney with a red arrow. **B** An axial pelvic CT-enhanced scan revealed a cystic low-density shadow approximately 50 mm in size located posterior to the bladder, along with a notably widened lower segment of the left ureter, as indicated by the red arrow. **C** A coronal CT plain scan showed both the cystic low-density shadow and the expanded ureter on the left side, as noted by the red arrow. **D** A sagittal CT plain scan delineated the cystic low-density shadow situated posterior to the bladder, marked by the red arrow

Ultrasound examination revealed an anechoic cystic mass (57.5 × 44.5 mm) adjacent to the left seminal vesicle, tracking along the mid-lower ureteral pathway (Fig. 1A-B). A contrast-enhanced computed tomography (CT) scan confirmed the absence of the left kidney, marked significant dilation of the left distal ureter (15 mm diameter), and identified a cystic low-density mass (50 mm diameter) posterior to the bladder, without evident enhancement (Fig. 2A-D).

**Fig. 3:**
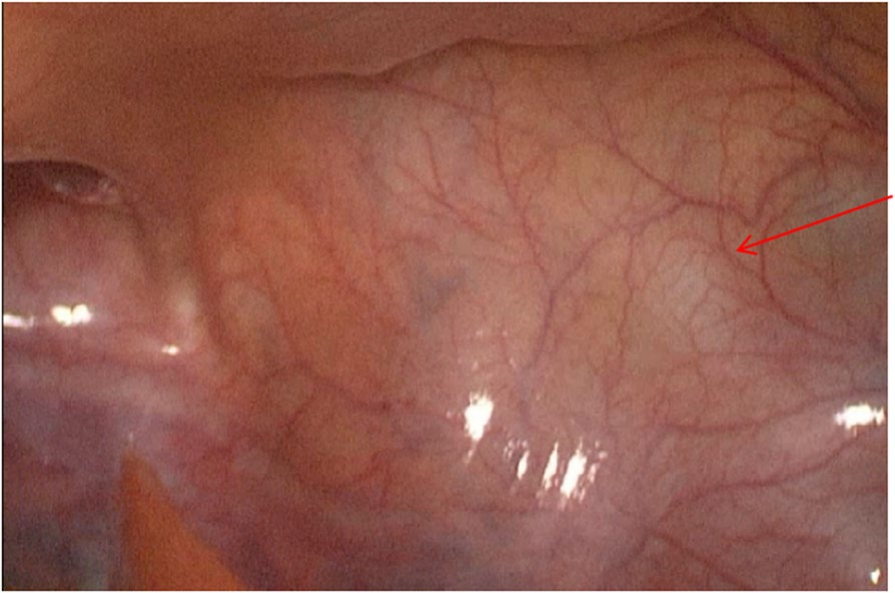
Laparoscopic findings of seminal vesicle cysts. A cystic mass, approximately 55 mm in size, was visible in the region of the left seminal vesicle (indicated by the red arrow)

**Fig. 4:**
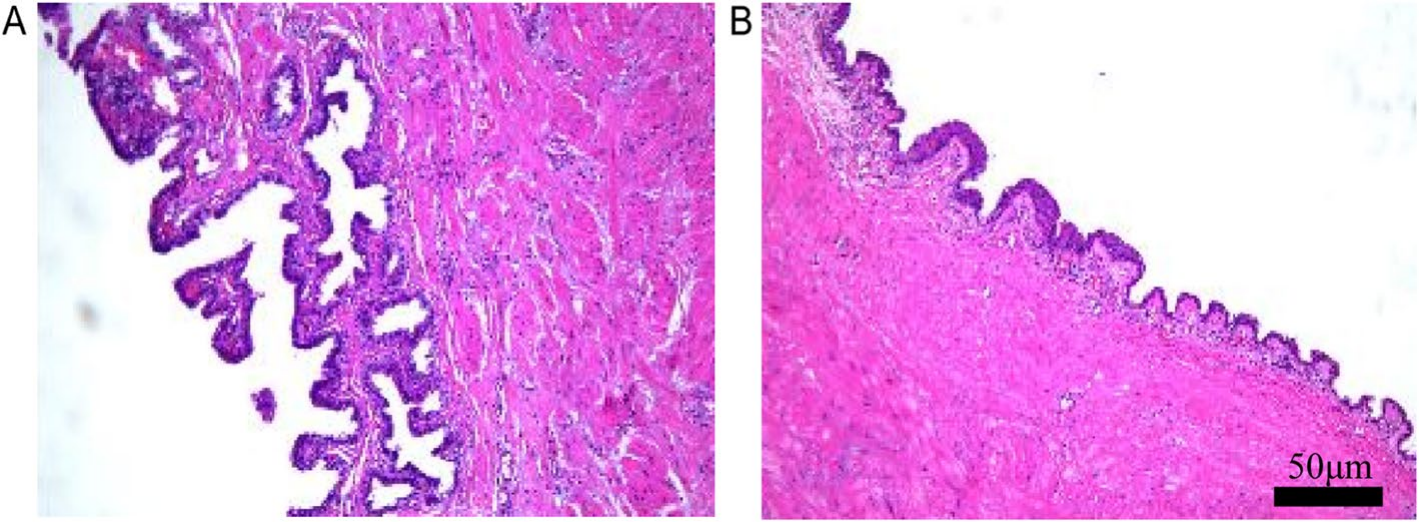
Microscopic examination of pelvic mass. **A**,** B** H&E staining: The microscopic pathological features aligned with those typically observed in a seminal vesicle cyst. Immunohistochemical staining results were as follows: GATA-Binding Protein 3 (*GATA 3*) ( +), Paired Box Protein 8 (*PAX 8*) ( +). Observations under a 20 × objective lens microscope, with scale bars = 50 µm

Following the exclusion of surgical contraindications, the patient underwent laparoscopic resection of the pelvic mass. Intraoperative exploration unveiled a 55 mm cystic mass adjacent to the left seminal vesicle (Fig. 3). The excised specimen displayed smooth-walled cystic morphology with intact inner lining. Postoperative histopathological analysis confirmed seminal vesicle cyst, corroborating preoperative diagnosis (Fig. 4A-B). The patient demonstrated significant alleviation of lower urinary tract symptoms following the procedure. On the fourth postoperative day, he was discharged from the hospital. At a six-month follow-up visit, the patient reported complete resolution of his lower urinary tract symptoms, indicating a successful surgical outcome.

To further investigate the etiology of the patient's condition, we performed a comprehensive WES analysis on both blood and tissue samples. The mean Q30 score exceeded 95%, with an average error rate was below 0.01%, indicating that the pre-processed data met the requisite quality standards. The transition/transversion ratio (Ts/Tv), a key indicator of single nucleotide polymorphism (SNP) dataset accuracy, was 2.51 in blood and 2.52 in tissue specimens. In blood and tumor tissue samples, 54,636 and 55,664 single nucleotide variants (SNVs) were identified, respectively (Supplementary Table 1). These SNVs were predominantly located in coding sequence (CDS) regions, introns, and 3'- untranslated regions (3'-UTRs) (Supplementary Fig. 1A, 2A). The predominant mutation types were synonymous and non-synonymous mutations (Supplementary Fig. 1B, 2B).

Somatic mutations refer to genetic alterations that occur in somatic cells, excluding germ cells. These mutations play a crucial role in understanding the initiation and progression of tumors. By excluding variants shared between tumor tissue and blood tissue, filtering out low-frequency variants, and removing variants with no clear functional impact, we identified 35 somatic SNVs with a variant allele frequency (VAF) greater than 5%. The genomic reference used in our analysis was GRCh37. All SNVs were annotated following the guidelines of the Human Genome Variation Society (HGVS) database (Supplementary Table 2) [8].

Due to the unavailability of parental DNA, we focused on rare homozygous variants. From this dataset, three genes were identified as potentially associated with Zinner syndrome (Table 1), all of which harbor rare homozygous variants. Specifically, *MYEOV* exhibited homozygous variants at three distinct loci, with the population frequency of the p.Leu302His variant being less than 0.001%. *BAGE* harbored two variants (one heterozygous, one homozygous) on chromosome 21. In *NAALAD2*, two homozygous variants were identified (Table 1).

**Table 1:** Screened Mutant Genes and Predisposing Genes Potentially Associated with Zinner Syndrome

Gene	position	Allele frequency	Nucleotide variation	Protein variation	Population frequency	Mutation type
MYEOV	GRCh37:11:69,063,822	0.064	c.905 T > A	p.Leu302His	< 0.001%	somatic variant
	GRCh37:11:69,063,984	1	NA^a^	NA	NA	germline variant
	GRCh37:11:69,063,572	1	c.655A > G	p.Met219Val	12.8%	germline variant
BAGE	GRCh37:21:11,059,982	0.091	NA	NA	NA	somatic variant
	GRCh37:21:11,059,877	0.475	NA	NA	NA	germline variant
NAALAD2	GRCh37:11:89,909,099	0.12	NA	NA	NA	somatic variant
	GRCh37:11:89,909,097	0.075	NA	NA	NA	somatic variant
NUTM1	GRCh37:15:34,635,863	0.464	c.30G > C	p.Lys10Asn	16.30%	somatic variant
	GRCh37:15:34,649,631	0.478	c.3338G > A	p.Arg1113His	10.80%	germline variant
	GRCh37:15:34,648,935	0.524	c.2642G > T	p.Ser881Ile	10.80%	germline variant
	GRCh37:15:34,638,342	0.566	NA	NA	NA	germline variant
	GRCh37:15:34,638,198	0.426	c.62A > G	p.Gln21Arg	14.60%	germline variant
PTPRD	GRCh37:9:8,518,052	0.503	c.1339C > G	p.Gln447Glu	4.41%	germline variant
	GRCh37:9:8,437,141	1	c.3989-452C > G	NA	7.93%	germline variant
	GRCh37:9:8,451,816	1	NA	NA	NA	germline variant
	GRCh37:9:8,451,817	1	NA	NA	NA	germline variant

This table lists screened mutant genes and predisposing genes potentially associated with Zinner Syndrome. For each gene, the following information is provided: chromosomal position (GRCh37), allele frequency, nucleotide variation, protein variation (if applicable), population frequency, and mutation type (somatic or germline). Variants with a population mutation frequency greater than 20% were excluded. Variants with no corresponding information in the database are marked as "NA". *MYEOV:* myeloma overexpressed, *BAGE:* B melanoma antigen, *NAALAD2:* N-Acetylated-Alpha-Linked Acidic Dipeptidase 2, *NUTM1:* NUT midline carcinoma family member 1, *PTPRD:* protein tyrosine phosphatase receptor type D

^a^NA indicates that no corresponding information for the specific variation was found in the database

**Fig. 5:**
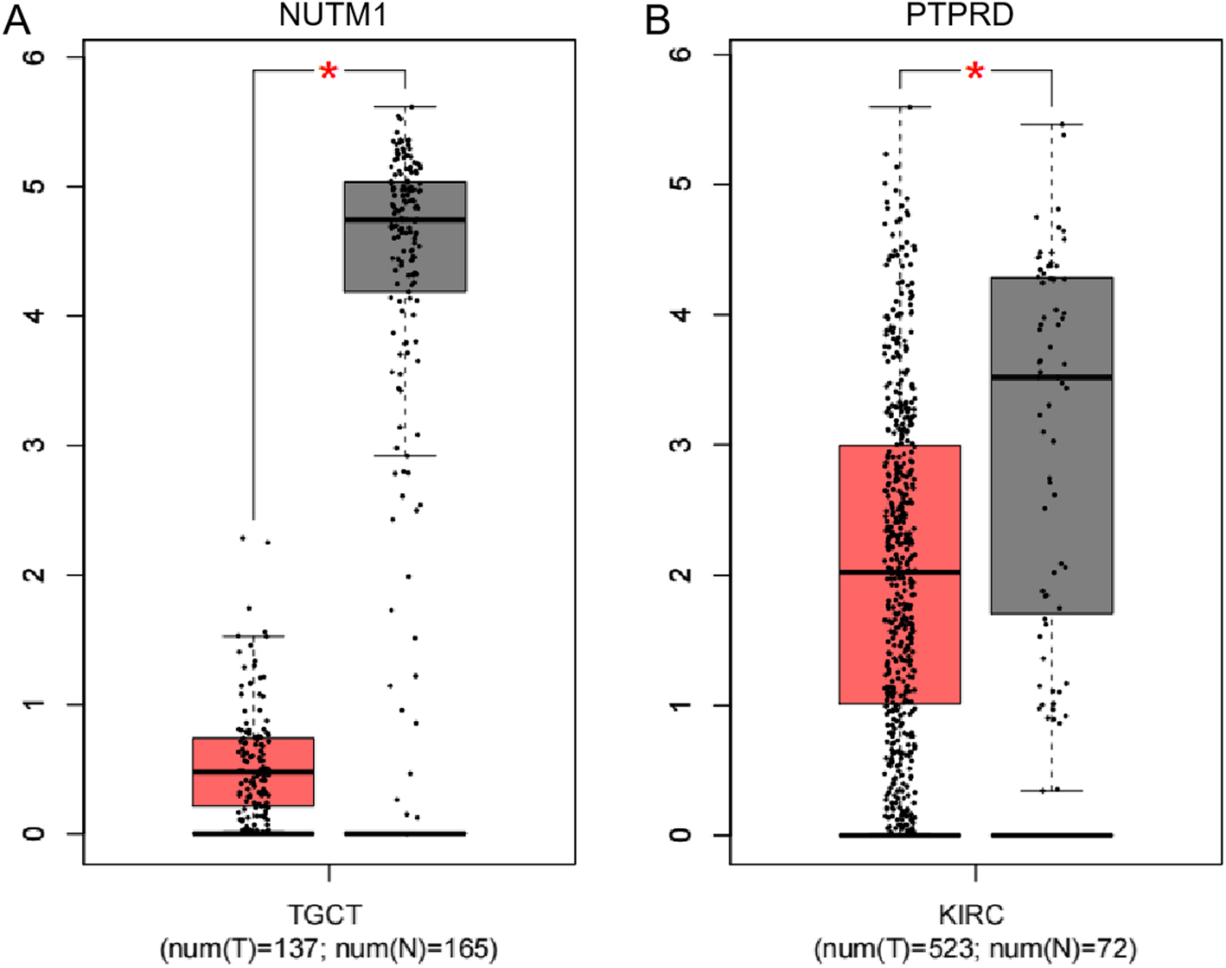
Differential gene expression of NUTM1 in TGCT and PTPRD in KIRC tumors analyzed by GEPIA. **A** Boxplot showing the downregulated expression of NUTM1 in TGCT tumors compared to normal tissues. **B** Boxplot showing the downregulated expression of PTPRD in KIRC tumors compared to normal tissues. TGCT, testicular germ cell tumors; KIRC, kidney renal clear cell carcinoma. Statistical differences were assessed by one-way ANOVA, with asterisks denoting significance levels (*P < 0.01). Red represents tumors, while gray represents normal tissues

Additionally, we compared the genetic variations detected in the patient's cells with the Cancer Gene Census (CGC) database, retained only genes with variant frequencies less than 20% in the general population, which led to the identification of 30 tumor-predisposing genes (Supplementary Table 3). Within this subset, focusing on rare homozygous or heterozygous variants, two genes were deemed rare and potentially relevant to Zinner syndrome: NUT midline carcinoma family member 1 (*NUTM1*) and protein tyrosine phosphatase receptor type D (*PTPRD*) (Table 1). These genes (*NUTM1*, *PTPRD*), identified through SNV analysis, were determined to be germline variants. *NUTM1* displayed heterozygous variants at five sites across chromosome 15. *PTPRD* displayed a heterozygous p.Gln447Glu variant, and the other three variants were homozygous variants (Table 1). *NUTM1* is a gene closely associated with NUT midline carcinoma (NMC) [9]. *PTPRD*, which is a receptor-type protein tyrosine phosphatase, plays a role in modulating the mitotic cycle and oncogenic transformation [10]. Through a search of the Gene Expression Profiling Interactive Analysis (GEPIA, http://gepia.cancer-pku.cn/) database, we demonstrated that the expression of *NUTM1* is decreased in testicular germ cell tumors (TGCT), while *PTPRD* shows marked downregulation in kidney renal clear cell carcinoma (KIRC) compared with normal tissues (Fig. 5A-B). However, through gene correlation prediction, no strong correlations were found among these two genes (Supplementary Fig. 3).

## Discussion

In this study, we report a case of typical Zinner syndrome in a young male, providing valuable genetic insights through WES of the patient's blood and tissue samples. By meticulously following established genetic sequencing protocols, as detailed in Appendix 1: Supplementary Methods, we ensured the accuracy and reliability of our results. Additionally, we present a comprehensive overview of this syndrome, which remains often under-explored within the context of andrological research.

Zinner syndrome was first described by A. Zinner in 1914 [11] and is typically observed in two-thirds of male patients with unilateral renal agenesis. To date, only approximately 200 cases have been reported in the literature [12]. The clinical manifestations of Zinner syndrome vary depending on the size of the seminal vesicle cyst, the degree of ejaculatory duct obstruction, and the duration of the disease. Generally, seminal vesicle cysts under 5 cm in size are asymptomatic [13]. However, as the cyst enlarges and begins to exert pressure on the bladder or other surrounding organs, symptoms including urinary urgency and frequency, dysuria, hematuria, perineal pain, and epididymitis may develop [14]. In cases of ejaculatory duct obstruction, patients could experience painful ejaculation, hemospermia, or even infertility [3, 12]. Additionally, a minority of patients may present with hydronephrosis or renal dysfunction in the healthy kidney, due to compensatory mechanisms prompted by lower urinary tract obstruction caused by the seminal vesicle cysts [15].

Current understanding suggests that disturbances of inductive processes, such as variations in metanephric blastema during the 4th to 7th weeks of gestation, can inhibit ureteric bud formation, leading to renal agenesis and atresia of the ejaculatory ducts [16]. Such disruptions may result in abnormal ureteral formation. Actually, the majority of these atypical and degenerated ureters may be ectopically inserted into other genitourinary structures, especially the seminal vesicles (22–33%) [12, 17]. Aberrant formation of the ejaculatory duct originating from the mesonephric system frequently leads to the accumulation of fluid internally, thereby culminating in seminal vesicle cysts [18]. The academic literature has recorded a rare case of clear cell carcinoma within the prostate, theorized to be attributable to embryonic renal remnants situated within or adjacent to the prostate [19]. These observations highlight the significant impact of embryonic developmental abnormalities in Zinner syndrome and the initiation and progression of tumors.

Mayer-Rokitansky-Küster-Hauser (MRKH) syndrome, often viewed as the female counterpart of Zinner syndrome, has been associated with LIM homeobox 1 *(LHX1),* hepatocyte nuclear factor 1 Beta *(HNF1B),* and Wnt family member 4 *(WNT4)* mutations in prior studies, which are critical for the development of the Wolffian duct [20]. Notably, while our WES analysis identified two *HNF1B* germline variants, these were excluded from further analysis as our study focused on somatic mutations underlying Zinner syndrome. No pathogenic variants were detected in *LHX1* and *WNT4*. This genetic divergence, compounded by phenotypic differences, reinforces distinct molecular mechanisms between these disorders. Inspired by the advance of WES in MRKH research, we focused on identifying novel Zinner-specific determinants [21]. Given that the impact of parental consanguineous marriage on the genetic interpretation has been ruled out, and in light of the unavailability of the parents' genetic data, our focus was concentrated on rare homozygous variants. Consequently, *MYEOV*, *BAGE*, and *NAALAD2* were selected as somatic-mutated genes and regarded as rare candidates exhibiting a potential correlation with Zinner syndrome.

*MYEOV* is a protein-coding gene associated with Myeloma [22]. Insights provided by GeneCards (https://www.genecards.org/) highlight associations between *MYEOV* and human phenotypes, as revealed through genome-wide association studies (GWAS), including links to prostate and kidney cancer. *BAGE* is known for its expression in the testes and a select group of malignant tumors, such as bladder cancer and melanoma, while being largely inactive in normal tissues [23]. This specific expression pattern makes *BAGE* a promising candidate gene for encoding tumor antigens [24]. In our study, genomic analysis revealed homozygous variants of the p.Leu302His variant in *MYEOV*. While the gnomAD v2.1.1 database reports a population frequency of < 0.001% for this locus, the gnomAD consortium cautions that this variant is covered in fewer than 50% of individuals in their exome dataset, rendering population frequency estimates unreliable. Given this technical limitation, we conservatively classify *MYEOV* as a candidate gene pending further validation, rather than definitively asserting its rarity. *NAALAD2*, belonging to the N-acetylated alpha-linked acidic dipeptidase (NAALADase) gene family, encodes the human prostate-specific membrane antigen (PSM), a recognized marker for prostatic carcinomas [25]. According to GeneCards, *NAALAD2* is expressed most prominently in the testes, a tissue specificity similar to that of *BAGE*. While the mutated genes identified through our WES analysis do not present direct evidence linking them to Zinner syndrome, their elevated expression levels in tissues and organs of the urogenital system, as well as their close association with diseases affecting this system, underscore the necessity for further investigation.

A predisposing gene is one that encodes hereditary diseases or increases disease susceptibility when exposed to appropriate environmental stimuli. Through comparison of variants identified in the patient's cells with the CGC database, we identified potential cancer susceptibility genes: *NUTM1* and *PTPRD*. *NUTM1* has a strong association with Nut midline carcinoma, a newly identified, rare, and highly aggressive squamous cell carcinoma characterized by extremely low survival rates [9]. Meanwhile, *PTPRD* is implicated in the progression of kidney cancer [26]. These susceptibility genes may be beneficial for the development of targeted preventive strategies for specific diseases. Despite mediating distinct biological mechanisms, these two genes are all closely associated with the occurrence and development of tumors, particularly urological tumors. Among numerous predisposing genes, they are the genes most likely to be linked to Zinner syndrome. However, in the absence of any evidence of Zinner syndrome within the family of the patient and the lack of reports on familial clustering of the disease in the extant literature, we are temporarily unable to speculate on whether the disease can occur through inheritance.

There have been documented instances where seminal vesicle adenocarcinoma and Zinner syndrome simultaneously occur in a patient [27]. The systematic review by Liu et al. highlighted several cases in which Zinner syndrome manifested as a postoperative comorbidity in urogenital cancer patients, along with documented co-occurrence with Kallmann syndrome – a rare neuroendocrine disorder [28]. It is speculated that Zinner syndrome may act as a contributing factor for the development of malignant tumors and may have complex interactions with various diseases [29].

Imaging technology has become increasingly pivotal in diagnosing Zinner syndrome. Ultrasound is particularly advantageous for screening Zinner syndrome due to its convenience and low cost, allowing for the detection of cystic masses in the pelvic cavity and providing preliminary assessments of renal status [30]. Abdominal and pelvic CT scans are valuable in reliably identifying ectopic kidneys that may not have fully developed [31]. MRI offers precise evaluation of all pelvic organs and surrounding soft tissue structures, aiding in distinguishing Zinner syndrome from other pelvic cysts, such as intraprostatic cysts, paramedian cysts, mimics of pelvic cysts, and bladder diverticula [31, 32].

Zinner syndrome is most commonly characterized by dysuria, frequent micturition, and perineal pain, comprising the classic triad mentioned above. Ultrasonography effectively determines the mass's location, size, and cystic nature, while MRI offers a more accurate diagnosis through analysis of cyst contents [33]. Our case presented with typical symptomatic Zinner syndrome, evidenced by perineal pain, urgency of micturition, and corroborative imaging findings. Furthermore, pathological investigations confirmed the diagnosis by excluding seminal vesicle adenocarcinoma and other conditions. Current consensus suggests conservative treatment for asymptomatic seminal vesicle cysts, while surgical resection is advocated for symptomatic cases [34]. In our case, surgical removal of the seminal vesicle cysts significantly alleviated the patient's pain symptoms and resulted in a favorable prognosis, underscoring the benefits of surgery for symptomatic cysts. Nevertheless, long-term follow-up is advised to ensure timely intervention in case of symptom recurrence or fertility issues [35].

Our study is subject to several limitations. Firstly, due to the rarity of Zinner syndrome, we included only a single case sample and conducted a single-center investigation, which necessitated a cautious interpretation of the genetic results. Attempts to acquire pertinent data from public databases, including GEO, were unsuccessful, as they contained no information on Zinner syndrome, which limits the generalizability of our genetic findings. Furthermore, due to personal considerations, the patient's parents opted not to participate in genetic testing, which hindered us from performing WES to explore potential genetic patterns related to Zinner syndrome. Consequently, only homozygous SNVs can be rigorously considered for analysis. It is equally imperative to acknowledge the inherent limitations of WES, notably its restricted coverage of non-coding areas, the variability in sequencing depth and potential for false positives or negatives. Lastly, with only half a year since the patient's surgery, we lack long-term follow-up data, which is another constraint of this study. Future research involving larger sample sizes and more extensive genetic analyses are essential to yield stronger insights into the pathophysiology of Zinner syndrome.

## Conclusion

Despite its rarity, Zinner syndrome can be effectively diagnosed via medical history, imaging, and histopathology. Insights into genetic mutations provide a deeper understanding of the etiological mechanisms, facilitating advanced exploration of prevention and treatment methods.

## Supplementary Information

Supplementary Material 1: Supplementary Table 1. The number of SNVs in different regions of the genome and in coding regions. The table summarizes the number of single nucleotide polymorphisms (SNPs) identified in tumor tissue and blood tissue across various genomic regions, including coding sequences (CDS), synonymous and nonsynonymous SNPs, stopgain and stoploss variants, intronic regions, untranslated regions (UTR3 and UTR5), splicing sites, non-coding RNA (ncRNA) regions, upstream and downstream regions, and intergenic regions. Variants with unknown functional impact are also included. The total number of SNPs in each tissue type is provided at the bottom of the table. Supplementary Table 2. Identification of 35 Genetic Variants through Somatic Single Nucleotide Variants (SNV) Analysis. The table lists 35 genetic variants identified through somatic SNV analysis. For each variant, the following information is provided: gene name, chromosomal position (GRCh37), allele frequency, nucleotide variation, protein variation (if applicable) and population frequency. Variants with no corresponding information in the database are marked as "NA". Supplementary Table 3. Identification of 30 Tumor-Predisposing Genes by Comparing Genetic Variations with the CGC Database. This table lists 30 tumor-predisposing genes identified by comparing genetic variations with the Cancer Gene Census (CGC) database. For each gene, the following information is provided: chromosomal position (GRCh37), allele frequency, nucleotide variation, protein variation (if applicable), population frequency, and associated cancer types from the CGC database. All screened genes have a population mutation frequency of less than 20%. Variants with no corresponding information in the database are marked as "NA". Supplementary Fig. 1. The pie chart shows the distribution of the number of single nucleotide variants (SNVs) in blood tissue. (A) The images show the number of SNVs in different regions of the genome. (B) The images depict the quantity of diverse mutation types of SNVs present within the coding regions. Supplementary Fig. 2. The pie chart shows the distribution of the number of single nucleotide variants (SNVs) in tumor tissue. (A) The images show the number of SNVs in different regions of the genome.(B) The images depict the quantity of diverse mutation types of SNVs present within the coding regions. Supplementary Fig. 3. Correlation analysis of NUTM1 and PTPRD gene expression analyzed by GEPIA. Scatter plot showing the negative correlation between NUTM1 and PTPRD expression (R = −0.1, *P* = 2.8 × 10 − 23). Although statistically significant, the correlation is weak, indicating no strong relationship between the expression levels of NUTM1 and PTPRD. This function performs pair-wise gene expression correlation analysis for given sets of TCGA expression data, using methods of Spearman.

## Data Availability

All data generated or analysed during this study are included in this published article and its supplementary information files.

## Appendix 1. Supplementary methods

### Quantification and quality assessment of DNA

Informed consent was obtained for all tissue and blood sample collections. Blood samples were collected during the patient's hospital stays, while tissue samples were obtained from surgical specimens. Both types of samples served for genomic DNA (gDNA) analysis.

Ensuring high-quality DNA is crucial for the accuracy of sequencing data. The quality of DNA was assessed using several methods: (1) DNA degradation was evaluated using agarose gel electrophoresis; (2) The sizing of genomic DNA fragments was analyzed with the Agilent 4200 TapeStation system (Agilent, Santa Clara, CA, USA); (3) DNA purity was assessed using the NanoDrop™ One/OneC spectrophotometer (Thermo Scientific, Waltham, MA, USA).

### Library construction for WES

The PCR product library was generated using the Agilent SureSelect Human All Exon V6 kit. Initially, 1 μg of genomic DNA was fragmented with dsDNA Fragmentase. This was followed by end-repairing, 3'-end A-tailing, and ligation with indexed adapters. Size selection was performed with Agencourt AMPure XP beads (Beckman Coulter Inc., Brea, CA). The DNA fragments were further processed for library construction using the KAPA Library Preparation kit (Kapa Biosystems, Inc., Wilmington, MA) according to the manufacturer’s protocol. Subsequent purification and quality analysis were conducted using the Agencourt AMPure XP beads and a NanoDrop™ One/OneC spectrophotometer. The DNA library was hybridized with biotin-labeled probes from the kit and captured with streptavidin-coated magnetic beads. Finally, the library was PCR-amplified and purified to obtain the WES library.

### Library quality analysis

The concentration of the constructed library was measured using the Qubit® 3.0 Fluorometer, and the molar concentration was determined by QuantStudio 3 qPCR. The Agilent 4200 system was utilized to examine the segment distribution within the library. Sequencing was then performed using the Illumina Novaseq 6000 system following the manufacturer's protocols.

### DNA-seq data processing, alignment, somatic mutation calling, and annotation

Raw DNA sequencing data underwent pre-processing with fastp (v0.18.0) to trim adapters, remove reads with excessive 'N' bases, and exclude low-quality bases, applying a threshold of ≤ 20 for more than 40% of the read length, as well as sliding window trimming. Reads were aligned to the reference genome (UCSC hg19) using Sentieon BWA with default settings. Subsequent processes, including sorting and duplicate removal, were handled by Sentieon driver. Depth and coverage metrics were derived using bamdst.

Sentieon DNAseq identified raw SNP/Indel sets, while somatic variations were detected using Mutect2. Somatic CNVs were identified with CNVkit, and somatic mutation features were extracted using the R package Sigminer. Finally, somatic mutation calls were annotated using ANNOVAR (version 2018-04-16).

## Abbreviations

BAGE: B melanoma antigen
CDS: Coding sequence
CGC: Cancer gene census
CNV: Copy number variations
CT: Computed tomography
GATA 3: GATA-binding protein 3
GEPIA: Gene Expression Profiling Interactive Analysis
GWAS: Genome-wide association study
HGVS: Human Genome Variation Society database
HNF1B: Hepatocyte nuclear factor 1-Beta
INDELL: Insertion and deletion
KIRC: Kidney renal clear cell carcinoma
LHX1: LIM homeobox 1
MRKH syndrome: Mayer-Rokitansky-Kustner-Hauser syndrome
MYEOV: Myeloma overexpressed
NAALAD2: N-acetylated-alpha-linked acidic dipeptidase 2
NUTM1: NUT midline carcinoma family member 1
PAX 8: Paired box protein 8
PTPRD: Protein tyrosine phosphatase receptor type D
PSM: Prostate-specific membrane antigen
SNP: Single nucleotide polymorphisms
SNV: Single nucleotide variant
TGCT: Testicular germ cell tumors
Ts/Tv ratio: Transition/transversion ratio
VAF: Variant allele frequency
WES: Whole exome sequencing
WNT4: Wnt family member 4
3'-UTRs: 3'-Untranslated regions
