# Supplementary material for: Zinner syndrome: report of a case and whole exome sequencing
Source: Basic Clin Androl. 2025 Mar 11;35:10. doi: 10.1186/s12610-025-00256-3 (PMC11895205; doi:10.1186/s12610-025-00256-3)
Supplement: Supplementary file 1 — Supplementary Material 1: Supplementary Table 1. The number of SNVs in different regions of the genome and in coding regions. The table summarizes the number of single nucleotide polymorphisms (SNPs) identified in tumor tissue and blood tissue across various genomic regions, including coding sequences (CDS), synonymous and nonsynonymous SNPs, stopgain and stoploss variants, intronic regions, untranslated regions (UTR3 and UTR5), splicing sites, non-coding RNA (ncRNA) regions, upstream and downstream regions, and intergenic regions. Variants with unknown functional impact are also included. The total number of SNPs in each tissue type is provided at the bottom of the table. Supplementary Table 2. Identification of 35 Genetic Variants through Somatic Single Nucleotide Variants (SNV) Analysis. The table lists 35 genetic variants identified through somatic SNV analysis. For each variant, the following information is provided: gene name, chromosomal position (GRCh37), allele frequency, nucleotide variation, protein variation (if applicable) and population frequency. Variants with no corresponding information in the database are marked as "NA". Supplementary Table 3. Identification of 30 Tumor-Predisposing Genes by Comparing Genetic Variations with the CGC Database. This table lists 30 tumor-predisposing genes identified by comparing genetic variations with the Cancer Gene Census (CGC) database. For each gene, the following information is provided: chromosomal position (GRCh37), allele frequency, nucleotide variation, protein variation (if applicable), population frequency, and associated cancer types from the CGC database. All screened genes have a population mutation frequency of less than 20%. Variants with no corresponding information in the database are marked as "NA". Supplementary Fig. 1. The pie chart shows the distribution of the number of single nucleotide variants (SNVs) in blood tissue. (A) The images show the number of SNVs in different regions of the genome. [file 12610_2025_256_MOESM1_ESM.docx]

Supplementary Material

# Supplementary Tables

Supplementary table 1. The number of SNVs in different regions of the genome and in coding regions.

| Sample | | Blood tissue | Tumor tissue |
| --- | --- | --- | --- |
| Coding Sequence (CDS) | Synonymous_SNP | 10,322 | 10,347 |
|  | Nonsynonymous _SNP | 9,304 | 9,364 |
|  | Frameshift_deletion | 90 | 88 |
|  | Frameshift_insertion | 71 | 70 |
|  | Nonframeshift_deletion | 120 | 119 |
|  | Nonframeshift_insertion | 123 | 120 |
|  | Stopgain | 70 | 71 |
|  | Stoploss | 8 | 9 |
|  | Unknown | 567 | 574 |
| Non-coding regions | Intronic | 24,968 | 2,5794 |
|  | UTR3 | 1,646 | 1,698 |
|  | UTR5 | 1,227 | 1,266 |
|  | Splicing | 122 | 131 |
|  | ncRNA_exonic | 1,593 | 1587 |
|  | ncRNA_intronic | 1,511 | 1,518 |
|  | ncRNA_UTR3 | 0 | 0 |
|  | ncRNA_UTR5 | 0 | 0 |
|  | ncRNA_splicing | 2 | 2 |
|  | Upstream | 571 | 597 |
|  | Downstream | 178 | 184 |
|  | Intergenic | 2,143 | 2,125 |
|  | Total | 54,636 | 55,664 |

Supplementary table 2. Identification of 35 Genetic Variants through Somatic Single Nucleotide Variants (SNV) Analysis.

| Gene | Position | Allele frequency | Nucleotide variation | Protein variation | Population frequency |
| --- | --- | --- | --- | --- | --- |
| ANKRD36C | GRCh37:2:96611013 | 0.178 | NA | NA | NA |
| GGT8P;ACTR3BP2 | GRCh37:2:92075610 | 0.136 | NA | NA | NA |
| ZDHHC11B | GRCh37:5:751455 | 0.268 | NA | NA | NA |
| UGT2B4 | GRCh37:4:70361154 | 0.069 | c.426G>A | p.Glu142Glu | <0.001% |
| MYEOV | GRCh37:11:69063822 | 0.064 | c.905T>A | p.Leu302His | <0.01% |
| TBC1D3P2 | GRCh37:17:60347151 | 0.113 | NA | NA | NA |
| PRIM2 | GRCh37:6:57433525 | 0.146 | NA | NA | NA |
| KRT6B | GRCh37:12:52843779 | 0.089 | c.816+17A>G | NA | 4.75% |
| AARS2 | GRCh37:6:44269660 | 0.05 | NA | NA | NA |
| PSG7 | GRCh37:19:43439413 | 0.072 | NA | NA | NA |
| CCDC144NL | GRCh37:17:20768931 | 0.128 | NA | NA | NA |
| FAM230G | GRCh37:22:20351704 | 0.192 | NA | NA | NA |
| NR5A2 | GRCh37:1:200080463 | 0.145 | c.1230+14C>T | NA | 35.3% |
| GK3P | GRCh37:4:166199009 | 0.122 | n.2167G>A | NA | 0.17% |
| LOC728613 | GRCh37:5:1629894 | 0.121 | NA | NA | NA |
| KMT2C | GRCh37:7:151935853 | 0.134 | NA | NA | NA |
| NBPF25P | GRCh37:1:145366463 | 0.119 | NA | NA | NA |
| NBPF19;NBPF20;NBPF8;NBPF9 | GRCh37:1:144823779 | 0.209 | NA | NA | NA |
| NBPF19;NBPF20 | GRCh37:1:144193482 | 0.127 | NA | NA | NA |
| ANKRD20A12P;LOC102723769 | GRCh37:1:142813200 | 0.07 | NA | NA | NA |
| ANKRD20A5P | GRCh37:18:14183974 | 0.052 | NA | NA | NA |
| NONE;GYG2P1 | GRCh37:Y:13524719 | 0.311 | NA | NA | NA |
| PABPC4L | GRCh37:4:135121567 | 0.098 | NA | NA | NA |
| DDX11L2 | GRCh37:2:114357350 | 0.237 | n.907A>G | NA | 34.5% |
| BAGE;BAGE2;BAGE3;BAGE4;BAGE5 | GRCh37:21:11059982 | 0.091 | NA | NA | NA |
| SLC25A46;TSLP | GRCh37:5:110284919 | 0.176 | NA | NA | NA |
| NAALAD2 | GRCh37:11:89909099 | 0.12 | NA | NA | NA |
| SFMBT2 | GRCh37:10:7262788 | 0.168 | NA | NA | NA |
| CCDC47 | GRCh37:17:61838197 | 0.076 | NA | NA | NA |
| TBC1D3P2;EFCAB3 | GRCh37:17:60374175 | 0.126 | NA | NA | NA |
| C6orf132 | GRCh37:6:42094588 | 0.161 | NA | NA | NA |
| CSMD2 | GRCh37:1:34631198 | 0.051 | NA | NA | NA |
| ARSH | GRCh37:X:2942330 | 0.113 | NA | NA | NA |
| GOLGA6L1;GOLGA6L22 | GRCh37:15:22743366 | 0.081 | NA | NA | NA |
| SDC1 | GRCh37:2:20401860 | 0.127 | NA | NA | NA |

**Note.** NA indicates that no corresponding information for the specific variation was found in the database.

Supplementary table 3. Identification of 30 Tumor-Predisposing Genes by Comparing Genetic Variations with the Cancer Gene Census (CGC) Database.

| Gene | Position | Allele frequency | Nucleotide variation | Protein variation | Population frequency | CGC_Cancers |
| --- | --- | --- | --- | --- | --- | --- |
| NTRK1 | GRCh37:1:156846320 | 0.541 | NA | NA | NA | papillary thyroid, Spitzoid tumour; |
| PTPN13 | GRCh37:4:87691080 | 0.403 | c.4663G>A | p.Val1555Ile | 00.036% | lung, NSCLC, gastric, peritoneal carcinomatosis;hepatocellular |
| NSD1 | GRCh37:5:176637576 | 0.509 | c.2176T>C | p.Ser726Pro | 20% | AML; |
| TRIP11 | GRCh37:14:92472416 | 0.348 | c.1904C>G | p.Ser635Cys | 1.43% | AML; |
| KMT2D | GRCh37:12:49434409 | 0.55 | c.7144C>T | p.Pro2382Ser | 1.19% | medulloblastoma, renal; |
| GOLGA5 | GRCh37:14:93263982 | 0.5 | c.200C>G | p.Ala67Gly | 10.3% | papillary thyroid, Spitzoid tumour; |
| AFF3 | GRCh37:2:100199386 | 0.43 | c.2742C>A | p.His914Gln | 0.37% | ALL, T-ALL; |
| ROS1 | GRCh37:6:117650532 | 0.516 | c.5326G>C | p.Asp1776His | 0.982% | glioblastoma, NSCLC, Spitzoid tumour, cholangiocarcinoma, borderline ovarian; |
| NUTM1 | GRCh37:15:34648935 | 0.524 | c.2642G>T | p.Ser881Ile | 10.8% | lethal midline carcinoma; |
| CLTCL1 | GRCh37:22:19183787 | 0.458 | c.4181T>C | p.Ile1394Thr | 6.42% | ALCL; |
| CRNKL1 | GRCh37:20:20033223 | 0.535 | c.247T>A | p.Ser83Thr | 5.94% | base cell carcinoma; |
| FAT1 | GRCh37:4:187542755 | 0.543 | c.4985A>G | p.Asn1662Ser | 15.5% | oral squamous cell, chemorefractory CLL, head and neck, pancreatic acinar cell carcinoma;pancreatic |
| RET | GRCh37:10:43615045 | 0.556 | NA | NA | NA | medullary thyroid, papillary thyroid, pheochromocytoma, NSCLC, Spitzoid tumour;medullary thyroid, papillary thyroid, pheochromocytoma |
| PER1 | GRCh37:17:8047081 | 0.6 | c.2575C>T | p.Pro859Ser | 1.91% | AML, CMML; |
| PTPRD | GRCh37:9:8518052 | 0.503 | c.1339C>G | p.Gln447Glu | 4.41% | lung cancer, kidney cancer, HNSCC, glioblastoma; |
| ARHGEF10 | GRCh37:8:1833801 | 0.991 | c.1110G>C | p.Leu370Phe | 15.4% | colon cancer; |
| CRTC1 | GRCh37:19:18876309 | 0.504 | c.1030A>G | p.Thr344Ala | 14.2% | salivary gland mucoepidermoid; |
| PDE4DIP | GRCh37:1:144931330 | 0.45 | NA | NA | NA | MPN; |
| PTPRB | GRCh37:12:70970293 | 0.534 | c.2711A>G | p.Gln904Arg | 1.6% | angiosarcoma; |
| PDGFRA | GRCh37:4:55147769 | 0.506 | NA | NA | NA | GIST, idiopathic hypereosinophilic syndrome, paediatric glioblastoma;GIST |
| CASC5 | GRCh37:15:40914177 | 0.333 | c.1793T>C | p.Met598Thr | 38.9% | AML; |
| RNF43 | GRCh37:17:56448297 | 1 | c.350G>A | p.Arg117His | 15.7% | cholangiocarcinoma, ovary, pancreas; |
| BRCA2 | GRCh37:13:32937526 | 0.354 | c.8187G>T | p.Lys2729Asn | 0.076% | breast, ovarian, pancreatic;breast, ovarian, pancreatic, leukaemia (FANCB, FANCD1) |
| TET2 | GRCh37:4:106155185 | 0.464 | c.86C>G | p.Pro29Arg | 6.05% | MDS; |
| CREB3L2 | GRCh37:7:137567348 | 0.5 | c.1297G>A | p.Glu433Lys | 0.025% | fibromyxoid sarcoma; |
| COL2A1 | GRCh37:12:48367976 | 0.509 | c.4213G>A | p.Gly1405Ser | 15.7% | chondrosarcoma, enchondroma; |
| USP8 | GRCh37:15:50773787 | 0.465 | c.1328A>G | p.Asp443Gly | 3.49% | corticotroph adenoma; |
| SIRPA | GRCh37:20:1896051 | NA | NA | NA | NA | HNSCC, colorectal cancer, Lung SCC; |
| MAP3K1 | GRCh37:5:56177848 | NA | NA | NA | NA | luminal A breast; |
| PRDM2 | GRCh37:1:14106394 | NA | NA | NA | NA | glioma, colon adenocarcinoma, gastric carcinoma, ovarian carcinoma, HNSCC; |

**Note.** NA indicates that no corresponding information for the specific variation was found in the database.

# Supplementary Figures


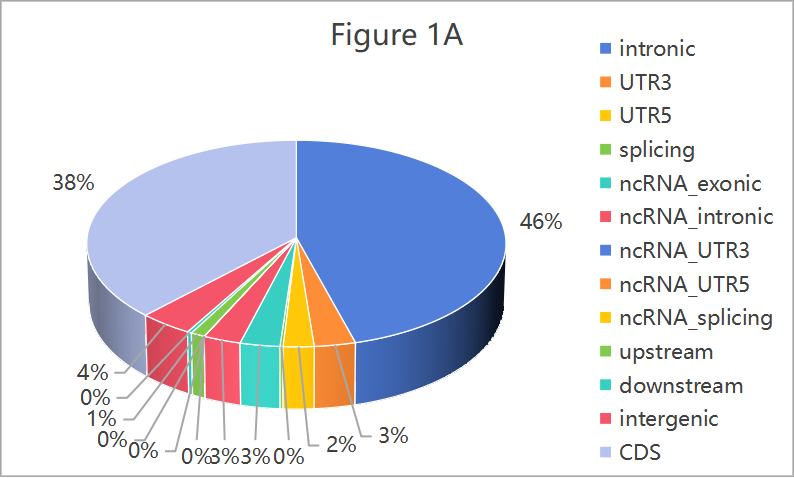

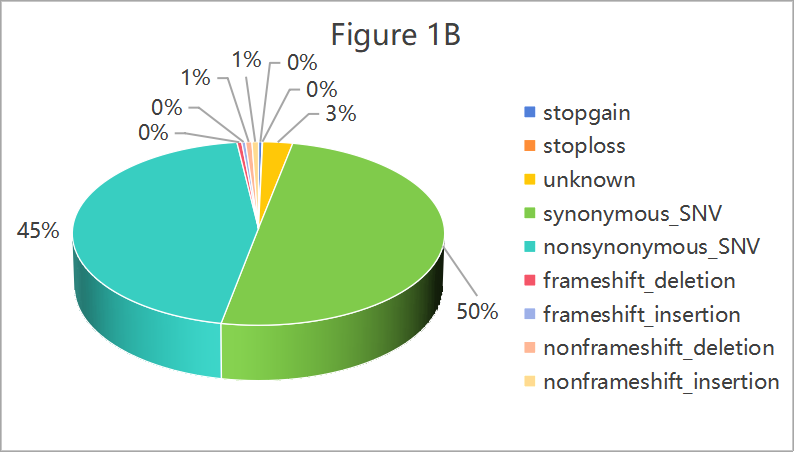


**Figure 1.** The pie chart shows the distribution of the number of single nucleotide variants (SNVs) in blood tissue. **(A)** The images show the number of SNVs in different regions of the genome.**(B)** The images depict the quantity of diverse mutation types of SNVs present within the coding regions.


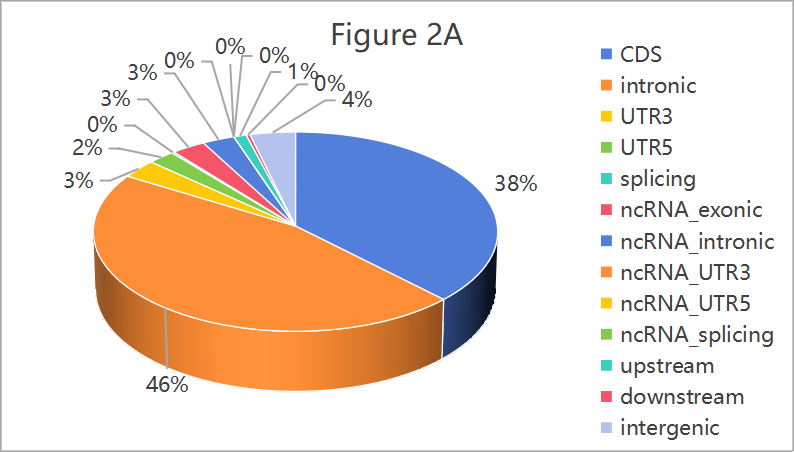

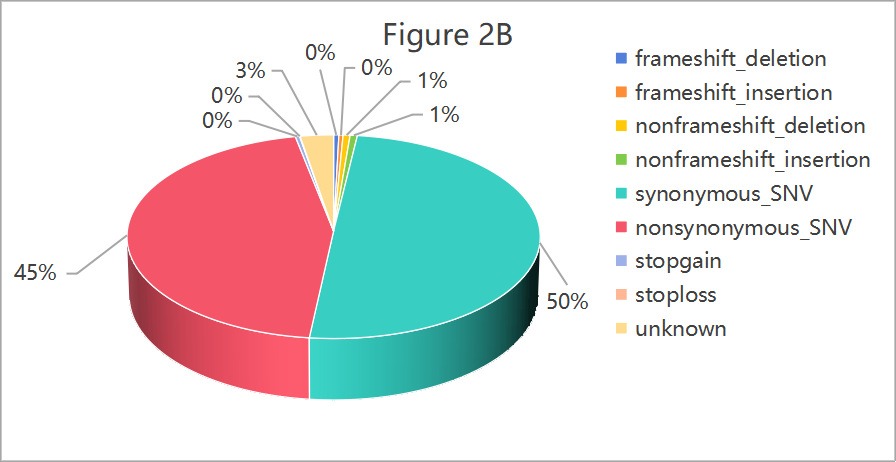


**Figure 2.**The pie chart shows the distribution of the number of single nucleotide variants (SNVs) in tumor tissue. **(A)** The images show the number of SNVs in different regions of the genome.**(B)** The images depict the quantity of diverse mutation types of SNVs present within the coding regions.


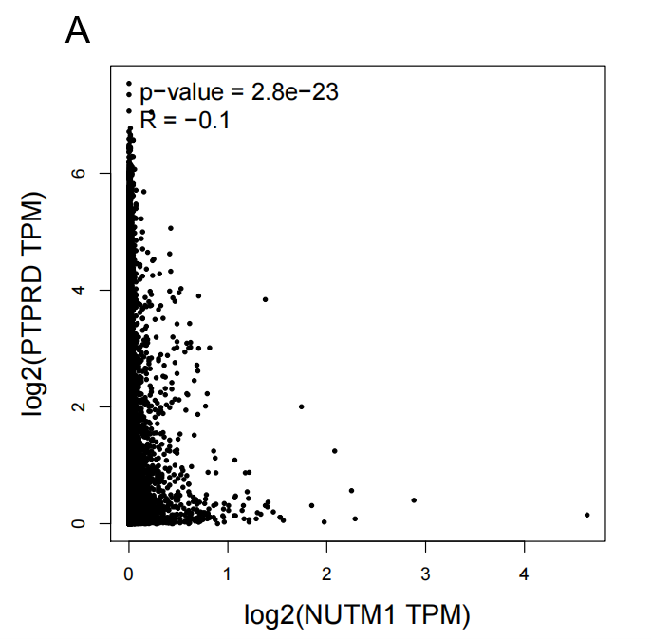


Figure 3. Correlation analysis of NUTM1 and PTPRD gene expression analyzed by GEPIA. Scatter plot showing the negative correlation between NUTM1 and PTPRD expression (R = -0.1, *P* = 2.8 × 10^−23^). Although statistically significant, the correlation is weak, indicating no strong relationship between the expression levels of NUTM1 and PTPRD. This function performs pair-wise gene expression correlation analysis for given sets of TCGA expression data, using methods of Spearman.
